# A phase IIb study to determine the safety and efficacy of candidate INfluenza Vaccine MVA-NP+M1 in combination with licensed Ina CTivated infl Uenza vaccine in adult S aged 65 years and above (INVICTUS): a study protocol

**DOI:** 10.12688/f1000research.19090.1

**Published:** 2019-05-23

**Authors:** Hannah Swayze, Julie Allen, Pedro Folegatti, Ly-Mee Yu, Sarah Gilbert, Adrian Hill, Chris Ellis, Christopher C. Butler

**Affiliations:** 1 Primary Care Health Sciences, University of Oxford, Oxford, UK; 2 The Jenner Institute, University of Oxford, Oxford, OX3 7DQ, UK; 3 Vaccitech Limited, Heatley Road, The Oxford Science Park,, Oxford, OX4 4GE, UK

**Keywords:** Influenza, vaccination, primary care, infection, MVA-NP+M1, older adults

## Abstract

Seasonal influenza has a significant annual global impact. Current influenza vaccines work by inducing strain-specific antibodies against the highly polymorphic surface proteins of the influenza virus and need to be redesigned every year, increasing their cost and limiting availability. There is a demand for a more efficacious vaccine, particularly in older adults in which the current vaccines show poor efficacy. The aim is to investigate a novel vaccine, MVA-NP+M1, which targets T cell responses to the nucleoprotein and matrix 1 core proteins of the influenza virus A, which are highly conserved, ****and therefore may provide long protection against a broad range of influenza strains.

INVICTUS is a phase IIb study to determine the safety and efficacy of candidate INfluenza Vaccine MVA-NP+M1 in combination with licensed Ina CTivated infl Uenza vaccine in adult S aged 65 years and above is a randomised, participant-blinded, placebo-controlled, multi-centre phase IIb efficacy study planned for 2030 volunteers aged 65 and over, in primary care. The primary objective is to assess the efficacy of MVA-NP+M1 co-administered with licensed inactivated quadrivalent influenza vaccine in adults ≥65 years. Participants complete daily diaries to record solicited and unsolicited events in the first four weeks post vaccination, and influenza-like illness (ILI) symptoms and severity throughout the influenza season.

We hypothesise an improvement in the primary outcome, a reduction in the average number of days spent with moderate or severe influenza-like illness during periods of influenza circulation, in the group administered with MVA-NP+M1, compared to those in the control group.

**Registration: **ClinicalTrials.gov identifier NCT03300362.

**Protocol version:** INVICTUS Protocol v3.0, 08 June06 2018.

## Abbreviations

AE, adverse event; CI, chief investigator; CRF, case report form; DMEC, data monitoring ethics committee; GCP, good clinical practice; GP, general practitioner; ICH, International Conference on Harmonisation; MHRA, Medicines and Healthcare products Regulatory Agency; MVA, modified vaccinia virus Ankara; NP, nucleoprotein; Pfu, plaque forming units; PIS, participant information sheet; QA, quality assurance; REC, research ethics committee; SAE, serious adverse event; SMPC, summary of medicinal product characteristics; SUSAR, suspected unexpected serious adverse reactions.

## Introduction

Seasonal influenza has a significant annual global impact accounting for an estimated 1 billion illnesses and 250,000–500,000 deaths ^1^ with an estimated economic cost of $87.1 billion in the US alone ^2^. The unpredictable risk of sporadic outbreaks of human infections with avian influenza (H5N1) could trigger a new pandemic if the virus acquires the ability to transmit from person to person ^3^. 

Vaccination remains the most cost-effective strategy available to combat influenza. Current influenza vaccines work by inducing strain-specific antibodies against the highly polymorphic surface proteins (haemagglutinin, neuraminidase) of the influenza virus. Vaccines are produced based on a prediction of strains likely to circulate in the population in the upcoming influenza season. The need for constant redesign and remanufacture increases cost and places limitations on vaccine supply ^4^, potentially leaving large populations susceptible to infection and illness. Efficacy of currently available influenza vaccines is significantly reduced in older adults (30–40%) compared to younger groups (70–90%) ^5^, which highlights the need for better protection in this age group.

When individuals are exposed to new influenza virus strains, to which they lack protective neutralising antibodies, cross-reactive T-cells against conserved internal antigens of influenza are associated with less viral shedding, reduced symptoms duration and severity ^6, 7^. We constructed MVA-NP+M1, a recombinant, replication-deficient modified vaccinia Ankara (MVA) vector expressing the conserved influenza antigens nucleoprotein (NP) and matrix 1 (M1) as a fusion protein ^8^, in the novel immortalised duck retinal cell line AGE1.CR.pIX. The aim of the vaccine is to boost cross-reactive T-cell responses to protective levels, providing immunity to not only human seasonal influenza, but also other influenza A subtypes currently found in avian species or swine.

Prior to INVICTUS, MVA-NP+M1 has been administered to 151 adults in seven clinical trials (see Table 1). The vaccine generally boosted T-cell responses when administered to healthy adults. In a small, un-randomised Phase IIa challenge study, fewer vaccinated volunteers developed influenza than unvaccinated ones and there was a trend toward a reduction in duration of virus shedding in vaccinated volunteers ^9^. The vaccine has been shown to have a good safety profile with no vaccine related serious adverse events (SAE) in these trials.

**Table 1.** Clinical studies to date using MVA-NP+M1 (Total n=151).

Country	Study	Vaccine	Age	Route	Dose of MVA- NP+M1	Number of volunteers
UK	FLU001	MVA-NP+M1	18–50	ID	5 ×10 ^7^ pfu	12
		MVA-NP+M1	18–50	IM	5 ×10 ^7^ pfu	8
		MVA-NP+M1	18–50	IM	2.5 ×10 ^8^ pfu	8
		MVA-NP+M1	50–59	IM	1.5 ×10 ^8^ pfu	10
		MVA-NP+M1	60–69	IM	1.5 ×10 ^8^ pfu	10
		MVA-NP+M1	70+	IM	1.5 ×10 ^8^ pfu	10
UK	FLU002	MVA-NP+M1	18–50	IM	1.5 ×10 ^8^ pfu	15
UK	FLU003	MVA-NP+M1 (together with seasonal influenza vaccine)	50+	IM	1.5 ×10 ^8^ pfu	9
UK	FLU004	ChAdOx1-NP+M1/MVA-NP+M1 ( *7–14 weeks apart*)	18–50	IM	1.5 ×10 ^8^ pfu	3
UK	FLU005	ChAdOx1-NP+M1 / MVA-NP+M1 *(8 weeks apart)*	18–50	IM	1.5 ×10 ^8^ pfu	12
		ChAdOx1-NP+M1 / MVA-NP+M1 *(52 weeks apart)*	18–50	IM	1.5 ×10 ^8^ pfu	8
		MVA-NP+M1 / ChAdOx1-NP+M1 *(8 weeks apart)*	18–50	IM	1.5 ×10 ^8^ pfu	13
		ChAdOx1-NP+M1 / MVA-NP+M1 *(52 weeks apart)*	18–50	IM	1.5 ×10 ^8^ pfu	12
		ChAdOx1-NP+M1 / MVA-NP+M1 *(8 weeks apart)*	>50+	IM	1.5 ×10 ^8^ pfu	12
UK	FLU006	MVA-NP+M1 ( *co-administered with seasonal* *influenza vaccine - Viroflu®*	>50	IM	1.5 ×10 ^8^ pfu	3
UK	FLU008	MVA-NP+M1 *(AGE1.CR.pIX cell line)*	18–50	IM	1.5 ×10 ^8^ pfu	6

## Protocol

### Methods and design

***Ethics and dissemination*.** The trial is conducted according to the principles of the Declaration of Helsinki, relevant regulations and good clinical practice, and will be disseminated through publication in peer-reviewed scientific journals. The study was approved by the South Central, Berkshire Research Ethics Committee (17/SC/0300).

### Objectives

The primary objective is to assess the efficacy of MVA-NP+M1 in combination with licensed inactivated influenza vaccine (IIV) in adults ≥65 years. See Table 3 for details of all objectives.

**Table 3.** Objectives, outcome measures and time-points.

Objectives	Outcome Measures	Timepoint(s) of evaluation and data collection method for this outcome measure (if applicable)
**Primary Objective** To assess the efficacy of MVA-NP+M1 in combination with licensed inactivated influenza vaccine in adults ≥65 years.	**Primary endpoint:** 1. Number of days with moderate or severe influenza-like symptoms	Throughout the influenza season - Self-reported symptoms recorded using electronic or paper diaries
**Secondary Objectives** To assess the incidence of ILI in adults aged 65 years and above vaccinated with MVA-NP+M1 or Placebo in combination with the recommended annual licensed inactivated vaccine.	1. Incidence of influenza-like-illness	Throughout the influenza season - Self-reported symptoms recorded using electronic or paper diaries
To assess the severity of influenza-like symptoms in adults aged 65 years and above vaccinated with MVA-NP+M1 or Placebo in combination with the recommended annual licensed inactivated vaccine	2. Severity of influenza-like symptoms	Throughout the influenza season - Self-reported symptoms recorded using electronic or paper diaries
To assess the duration of ILI in adults aged 65 years and above vaccinated with MVA-NP+M1 or Placebo in combination with the recommended annual licensed inactivated vaccine	3. Duration of influenza-like-illness	Throughout the influenza season - Self-reported symptoms will be recorded using electronic or paper diaries
To assess the occurrence of GP consultations from respiratory illness in adults aged 65 years and above vaccinated with MVA-NP+M1 or Placebo in combination with the recommended annual licensed inactivated vaccine	4. Occurrence of GP consultations from respiratory illness	Throughout the influenza season - Self-reported and Medical Records
To assess the hospitalisations and deaths due to respiratory illness in adults aged 65 years and above vaccinated with MVA-NP+M1 or Placebo in combination with the recommended annual licensed inactivated vaccine	5. Occurrence of hospitalisations and deaths due to respiratory illness	Throughout the influenza season - Self-reported and Medical Records
To assess the safety and reactogenicity of MVA-NP+M1 in combination with licensed inactivated influenza vaccine in adults ≥65 years. To assess the immunogenicity of MVA-NP+M1 or Placebo in combination with the recommended licensed inactivated influenza vaccine in adults aged 65 years and above To assess the presence of the influenza virus using virological markers, in adults aged 65 years and above vaccinated with MVA- NP+M1 or Placebo in combination with the recommended annual licensed inactivated vaccine	6. Occurrence of solicited local and systemic reactogenicity signs and symptoms for 7 days following vaccination 7. Occurrence of unsolicited adverse events for 28 days following vaccination 8. Occurrence of serious adverse events throughout participants’ participation in the trial - Frequency of influenza-specific T-cells measured by IFN-γ ELISpot - Geometric mean titre of influenza-specific neutralising antibodies - Breadth of influenza-specific T-cells and antibodies 1. Incidence rate of laboratory confirmed influenza using RT-PCR.	Day 0–7 - Self-reported symptoms recorded using electronic or paper diaries Day 0–28 - Self-reported symptoms recorded using electronic or paper diaries Telephone calls on Day 1–3, day 7–9 and every 3–4 weeks throughout participants’ participation in the trial Blood samples drawn at enrolment (before vaccinations), day 21 and at the end of the influenza season Nasal swab sample taken by participant at the time of self-reported symptoms. Nasal swabs will be taken during the first 3 days of the onset of the self-reported symptoms
**Tertiary Objectives** To explore novel clinical endpoints for future Phase III efficacy trials of influenza vaccines	1. Estimated frequency of influenza infection using historical data on the proportion of ILIs that is caused by influenza virus infection.	At the end of the influenza season

### Study design

The trial is an individually randomised, participant-blinded, placebo-controlled, multi-centre phase IIb efficacy study in 2030 volunteers aged 65 and over, in England. The main study is set in primary care and a separate immunology sub-cohort of 100 participants will be recruited at the Jenner Institute, University of Oxford. The trial will be promoted through various media sources.

### Interventions

Participants are randomly assigned to either the control (licensed IIV and placebo) or intervention group (licensed IIV and MVA-NP+M1) by the Research Nurse.

***MVA-NP+M1*.** The vaccine has been described previously and consists of a replication deficient MVA viral vector expressing the NP and M1 antigens from the influenza A virus (H3N2, 87 A/Panama/2007/99) as a single fusion protein ^8^. A dose of 1.5 ×10 ^8^ pfu will be used. MVA-NP+M1 is manufactured under good manufacturing practice conditions by Emergent Biosolutions, USA, and is certified and labelled for trial at the Clinical Biomanufacturing Facility, University of Oxford. The vaccine is stored at -80°C in a temperature monitored, secure freezer at the clinical sites.

***Seasonal influenza vaccine (licensed IIV)*.** Pre-packaged needle and syringe containing 0.5 ml licensed IIV (Split Virion) is stored at 2°C to 8°C in a secure, temperature-monitored refrigerator at site. 

***Placebo*.** Participants allocated to the control group will receive an intramuscular injection of 0.9% saline. The volume and site of injection will be the same as for the MVA-NP+M1 group.

### Trial procedures

***Informed consent*.** Once eligibility has been confirmed ( Table 2), participants provide written informed consent before any study procedures are performed. The participant is fully informed of all aspects of the trial, the potential risks and their obligations, and will be able to ask questions.

**Table 2.** Eligibility criteria.

Inclusion Criteria	Exclusion Criteria
• Volunteer is willing and has capacity to provide written informed consent for participation in the trial (in the Investigator’s opinion). • Male or female adults, aged 65 years and above • Able and willing (in the Investigator’s opinion) to comply with all study requirements • Willing to allow the investigators to discuss the volunteer’s medical history with their General Practitioner • Eligible to receive seasonal influenza vaccine	• Any history of anaphylaxis in reaction to vaccination or history of allergic reactions likely to be exacerbated by any component of the vaccine (e.g. egg allergy) • Ongoing terminal illness with a life expectancy estimated to be approximately <6 months. • Continuous use of oral anticoagulants, such as coumarins and related anticoagulants (i.e. warfarin) or novel oral anticoagulants (i.e. apixaban, rivaroxaban, dabigatran and edoxaban) • Any other significant disease, disorder or finding (including blood test results), which, in the opinion of the Investigators, would either put the volunteer at risk because of participation in the study, or may influence the result of the study • Participation in another clinical trial of an investigational medicinal product in the 30 days preceding enrolment, or planned use during the study period • Prior receipt of an investigational vaccine likely to impact on interpretation of the trial data • Receipt of annual seasonal influenza vaccine prior to enrolment (for the same influenza season volunteers are recruited in) • Not willing to comply with study procedures

***Vaccination procedure*.** The seasonal influenza vaccine is administered first, and then saline or MVA-NP+M1 administered no longer than 5 minutes after, within a 1cm circle. All vaccines are administered intramuscularly into the deltoid region of the arm. 

***Participant observation*.** Participants are observed for a minimum of 10 minutes after vaccination. Medicines and resuscitation equipment are available for the management of anaphylaxis. Participants are provided with an oral thermometer, follow-up diaries and a card with study contact details. An out of hours telephone number is provided.

***Vaccination postponement*.** Vaccination may be postponed in any of the following events at the time of vaccination.

- Acute disease
- Temperature of >37.5°C.
- Receipt of a licensed inactivated vaccine within 2 weeks prior to enrolment.
- Receipt of a licensed live vaccine within 4 weeks prior to enrolment.

### Sub-cohort

A sub-cohort of participants (50 for each of two seasons) will be recruited to assess the immunogenicity of MVA-NP+M1 in combination with the licensed IIV. In addition to the procedures described for the main cohort, the sub-cohort attend a screening visit, and three further visits where blood samples are taken for monitoring of laboratory AEs and immunology purposes at weeks 1, 3 and 26. Blood samples will be stored in compliance with the Human Tissue Act (2004).

### Follow-Up procedures

***Participant Diaries*.** Participants record their symptoms in electronic diaries or paper diaries. Daily or weekly reminders are sent.

**Week 1 diary**. Participants are asked to record the occurrence and severity of solicited and unsolicited adverse events for one week. The following solicited adverse events are recorded by participants: local (pain, redness, warmth and pruritus at injection site) and systemic (feverishness, chills, myalgia, fatigue, headache, nausea, vomiting, arthralgia, malaise and oral temperature). 

**Week 2–4 diary**. From weeks 2–4, participants report unsolicited adverse events, and influenza-like illness (ILI): feverishness, cough, sore throat, malaise, headache, myalgia, shortness of breath, runny/blocked nose, sneezing and temperature. For the duration of ILI symptoms they record: the severity of symptoms (mild, moderate or severe), oral temperature and any new medication.

**Daily and symptom diary**. From week 5 until the end of the flu season, participants are only required to report ILI as described for weeks 2–4.

***Safety follow-up*.** Participants are contacted at 24 (+48 h) hours, on day 7 (+2 days) and every 3–4 weeks after vaccination, to monitor and record the occurrence of any SAEs and GP consultations, remind participants to complete their diary and to collect ILI data when a diary hasn’t been completed.

### Medical notes review

A medical notes review is conducted to collect details of any occurrences in the follow-up period of: laboratory-diagnosed influenza illness, medically attended respiratory illness, GP consultations, antibiotic prescriptions and hospitalisations.

### Data management

Details can be found in the Data Management Plan, held at the PC-CTU, University of Oxford. The plan is also available as *Extended data* ^10^.

***Confidentiality*.** Participants will be identified only by a participant ID number. The trial will comply with the Data Protection Act 2018. All documents will be stored securely and only accessible by trial staff and authorised personnel, at the University of Oxford.

***Access to data*.** Vaccitech Ltd. and the University of Oxford will have access to the final trial dataset. GP sites will have access to the dataset for participants registered at their practice. Access to the data will be outlined in the relevant contract.

### Outcomes

***Primary endpoint*.** The primary endpoint is the number of days with moderate or severe ILI during the influenza season defined using UK surveillance data. A protocol ILI definition will be adopted for the purposes of analysis: feeling feverish or having a fever (temperature ≥37.8°C) and at least one of the following symptoms: a cough and/or sore throat. See Table 3. for details of all primary, secondary and tertiary endpoints.

### Randomisation

The study team obtain the randomised group allocation through a validated and secured web-based server (Sortition®). Randomisation will use non-deterministic minimisation on practice, age and gender to ensure each arm is balanced and 1:1 allocation when all participants have been recruited. The immunology sub-cohort will be randomised separately using the same method. Emergency randomisation procedure is also available if the web-based system isn’t accessible.

### Blinding

The participant is blinded to the group they have been allocated to, but the staff administering the vaccine aren’t blinded. Investigators recording and assessing clinical and safety outcomes are blinded to group allocation.

### Sample size

We assumed the average number of days spent with moderate or severe ILI per “season” of circulation was 3.5 days and a typical follow-up period for a season of approximately 120 days across this corresponds to 2.92% of days [6]. A sample size of 2,030 (1,015 per group) will provide 85% power to detect a relative drop of 20% in the primary outcome between the two groups (from 2.92% to 2.34%) at 5% level of significance (2-sided). This sample size has accounted for a 25% attrition rate and a further 15% increase to account for clustering of participants within households, which are estimated to be 0.04.

For the secondary outcomes, we estimated that 12.25% of vaccinated individuals over 65 years old experience an ILI most likely attributable to influenza [6].The proposed sample size will have 90% power to a reduction in the percentage of individuals experiencing ILI from 28.5–22.25%. We estimate a 50% improvement in vaccine efficacy over the IIV alone in reducing the occurrence of ILI due to influenza during periods of influenza circulation.

### Statistics

***Description of statistical methods*.** The primary statistical analysis will be by intention-to-treat (ITT). Between group comparisons will be presented using effect measures (ratio or difference in response rate, difference in means) with 95% confidence intervals.

The primary outcome will be modelled using a generalised linear mixed effect logistic model, adjusting for flu season (first or second season), age, sex, GP practice and chronic illnesses. The outcome will be presence of any of the listed symptoms (feverishness, cough, sore throat, malaise, headache, myalgia, shortness of breath and temperature) rated as moderate or severe by participants who meet the protocol ILI definition on any of the days that are included for study purposes. Random effects will be incorporated to account for the clustering of participants within households and to account for the clustering of data over days within participant.

Comparison between groups of the proportion of participants experiencing an ILI will be carried out using the same method as the. The mixed effects will acknowledge the clustering of participants within households. The effect measure presented for MVA-NP+M1 compared to IIV alone will be an adjusted odds ratio with 95% confidence interval, which can be interpreted as an adjusted relative risk given the low prevalence.

We will use mixed-effect model, or an equivalent nonparametric method where appropriate, to analyse continuous secondary outcomes, adjusting for the same baseline measures and stratification variables. We will compare the safety outcomes using Chi-squared or Fisher’s exact test.

All the tests will be done at a 5% two-sided significance level. Data analysis will be restricted to the periods where acute respiratory illnesses are likely to be attributable to influenza based on local and national surveillance intelligence.

***Immunogenicity analyses*.** In the immunology sub-cohort, the primary analysis will be to assess the difference in magnitude of influenza-specific T-cell (using interferon gamma enzyme-linked immunospot assay) and antibody responses (using influenza hemagglutination inhibition assay) between the two groups. We will assess the vaccine immunogenicity by comparing the change in these immunological parameters from baseline to 21 days after vaccination between the groups, and how this varies according to baseline factors of age, gender and chronic illness. To assess the durability of immune responses post-vaccination, the stability of immunological parameters between the post vaccine time point and end of season time point will be assessed. The immunological x will be log transformed (based 10) and we will use appropriate regression or correlation methods to carry out the analysis.

***Reporting procedures for adverse events*.** All adverse events (AEs) occurring in the first 28 days post-vaccination are captured in the diaries. The relationship of each AE to the trial medication is determined by a medically qualified individual. All AEs that result in a patient’s withdrawal from the study will be followed up until a satisfactory resolution occurs, or a non-study related causality is assigned. All reported severe symptoms are reviewed by the Medical Monitor in the first 4 weeks.

***Reporting procedures for SAEs*.** All SAEs are documented and reported according to the Pharmacovigilance SOP. The assessment of expectedness is made by a clinician using the reference safety information current at the time of the event. If deemed necessary un-blinding will occur in accordance with trial specific working instructions.

***Group holding rules*.** If a group holding rule is activated, further vaccinations won’t occur until it is deemed appropriate to restart, by the MHRA: if more than 30% of vaccinations are followed by the same Grade 3, solicited local AE or solicited systemic AE or unsolicited AE, beginning within 2 days after vaccination and persisting at Grade 3 for >48 hrs. Other events that trigger the rule include related death, life-threatening reaction, SUSAR, acute allergic reaction or anaphylactic shock. The study can also be put on hold upon advice of the Chief Investigator, Sponsor, MHRA, REC or DMEC.

### Discontinuation/withdrawal

Participants may withdraw voluntarily, on the decision of the Investigator or on the advice of the DMEC. If a participant withdraws from the study, data and blood samples collected before their withdrawal will be used/stored unless they specifically requests otherwise.

### Monitoring

Regular monitoring at investigator sites is performed according to ICH GCP and a monitoring plan.

### Sponsor and funder

This study is sponsored and funded by Vaccitech Ltd.

### Joint Data Monitoring and Ethics Committee and Trial Steering Committee

A joint DMEC and Trial Steering Committee periodically reviews and evaluates the accumulated study data for participant safety, study conduct and progress, and efficacy. The committee includes three appropriately qualified members. The DMEC will review SAEs deemed at least possibly related to study interventions.

### Interim analyses

Three interim safety reviews are planned to evaluate the safety data accumulated up to the following time points (by the DMEC):

1. After the first 100 participants have been randomised
2. The end of vaccinations in the first year of the study
3. The end of the 1 ^st^ year of follow-up

## Discussion

There is an urgent and pressing demand for improved vaccination strategies against a broad spectrum of influenza virus strains. INVICTUS aims to investigate whether MVA-NP+M1 is capable of providing protection against a broad spectrum of influenza A virus strains with better immune responses in older people, who are at higher risk of severe influenza disease.

INVICTUS is the first clinical trial to investigate whether the combination of MVA-NP+M1 and the seasonal vaccine, reduces ILI symptoms in patients, which would be both clinically useful and considerable step forward in vaccine development. 

## Data availability

### Underlying data

No underlying data are associated with this article.

### Extended data

Open Science Framework. F1000Research INVICTUS Protocol Manuscript. https://doi.org/10.17605/OSF.IO/T6P2B ^10^.

Extended data are available under the terms of the Creative Commons Zero "No rights reserved" data waiver (CC0 1.0 Public domain dedication).

### Reporting checklists

SPIRIT checklist for “A phase IIb study to determine the safety and efficacy of candidate INfluenza Vaccine MVA-NP+M1 in combination with licensed InaCTivated inflUenza vaccine in adultS aged 65 years and above (INVICTUS): a study protocol”. https://doi.org/10.17605/OSF.IO/T6P2B ^10^.
