# Supplementary material for: A phase IIb study to determine the safety and efficacy of candidate INfluenza Vaccine MVA-NP+M1 in combination with licensed Ina CTivated infl Uenza vaccine in adult S aged 65 years and above (INVICTUS): a study protocol
Source: F1000Res. 2019 May 23;8:719. [Version 1] doi: 10.12688/f1000research.19090.1 (PMC7014788; doi:10.12688/f1000research.19090.1)
Supplement: Peer review history

# Peer review: Reviewer response for version 1

**DOI:** 10.5256/f1000research.20920.r51322

**Published:** 2020-02-11

**Authors:** Rebecca J. Cox

**Affiliations:** 1 Influenza Centre, Department of Clinical Science, University of Bergen, Bergen, Norway

This report is a study protocol for the INVICTUS clinical trial which is a phase IIb study to evaluate the safety and efficacy of seasonal IIV in combination with MVA_NP+M1. The protocol is well written and comprehensive. 

 There are a couple of minor issues which need to be addressed:

**Abstract:** long protection should perhaps be changed to long term protection.

**Page 5:** the geometric titres of neutralising antibodies are listed as outcome measures, this should be changed to HAI titres as the protocol only specifies that HAI tests will be conducted. If neutralising titres are also measured by MN or vN assays this can remain.

**Page 7:** this sentence is incomplete; comparison between groups of the proportion of participants experiencing an ILI will be carried out using the same method as **the.**

**Page 7:** it is not entirely clear what immunological x is; the **immunological x **will be log transformed (based 10) and we will use appropriate regression or correlation methods to carry out the analysis.

I confirm that I have read this submission and believe that I have an appropriate level of expertise to confirm that it is of an acceptable scientific standard.

# Peer review: Reviewer response for version 1

**DOI:** 10.5256/f1000research.20920.r54948

**Published:** 2020-01-24

**Authors:** Xavier Saelens

**Affiliations:** 1 Department of Biomedical Molecular Biology, Ghent University, Ghent, Belgium

The manuscript describes the study protocol of a planned phase IIb study (INVICTUS) that aims to determine the safety and efficacy of an MVA vectored influenza NP-M1 fusion construct combined with a conventional influenza vaccine in adults aged 65 years or older. The conventional influenza vaccine (split virion) will be administered by standard intramuscular injection followed, within 5 minutes, by saline (placebo) or MVA-NP + M1 vaccine, also injected intramuscularly close to the influenza vaccine injection site. The trial intends to recruit 2030 volunteers, 1:1 randomized over the 2 study arms. The study will for the most be conducted in primary care settings in the UK. An immunology sub-cohort of 100 volunteers is included as well for the monitoring of seroconversion (influenza hemagglutination inhibition) and cellular responses (ELISPOT). The primary outcome is to assess the efficacy of MVA-NP + M1 combined with a conventional inactivated influenza vaccine in terms of the number of days the volunteers experience moderate or severe influenza-like symptoms based on a self-reported symptoms. Secondary outcomes include comparison of the occurrence of hospitalization and death due to respiratory illness, the rate of laboratory confirmed influenza (based on RT-PCR) and the magnitude of the immune responses.

 In a WHO bulletin published in November 2017, it is recommended to omit “sore throat” from the clinical case definition of influenza-like illness. This may be taken in consideration for the primary endpoint determination.

 The peptides that will be used to determine the magnitude of the influenza-specific T cell response should be specified. Only NP and M1-derived peptides? Corresponding to the Panama/99 virus or also to recently circulating influenza viruses? The analysis of the breadth of the antibody responses could be better defined.

 The statement on influenza vaccine efficacy in older adults should be supported by more recent references (e.g. Beyer et al. Vaccine, 2013 ^1^; Demicheli et al., Cochrane DSR, 2018 ^2^).

 The first sentence of the 3 ^rd^ paragraph under Statistics is incomplete.

I confirm that I have read this submission and believe that I have an appropriate level of expertise to confirm that it is of an acceptable scientific standard.

## References

[1] : Cochrane re-arranged: support for policies to vaccinate elderly people against influenza. *Vaccine*.2013;31(50) : 10.1016/j.vaccine.2013.09.063 6030-3 10.1016/j.vaccine.2013.09.063 24095882

[2] : Vaccines for preventing influenza in the elderly. *Cochrane Database of Systematic Reviews*.2018; 10.1002/14651858.CD004876.pub4 10.1002/14651858.CD004876.pub4 PMC649110129388197

# Peer review: Reviewer response for version 1

**DOI:** 10.5256/f1000research.20920.r53395

**Published:** 2019-09-24

**Authors:** Tamar Ben-Yedidia

**Affiliations:** 1 BiondVax Pharmaceuticals Ltd., Ness Ziona, Israel

The manuscript describes a Ph2b clinical trial to evaluate the safety and efficacy of a universal vaccine candidate against influenza. The novel vaccine is a fusion protein of MVA, with NP and M1 viral proteins that are conserved and hence, are expected to provide broad cross strain protection. The vaccine candidate activates cell mediated immunity. The trial focuses on the at-risk elderly group that will be administered once with the experimental vaccine together with the licensed inactivated vaccine against flu. A sub cohort will be tested for immunogenicity.

 Main study: There are multiple viruses that cause ILI (other than Influenza) that are not expected to be affected by this vaccine. It is not clear why the sponsor expects to have reduced influenza like illness by immunizing against Influenza. Why they do not confirm Influenza illness (e.g. by PCR) and evaluate the reduction of illness in this sub population.

 Sub cohort: The specificity of the immunological parameters is not described. Do they plan to evaluate the response to the vaccine Ags? or to different influenza viruses/antigens? How will they show the broad response to the vaccine?

I confirm that I have read this submission and believe that I have an appropriate level of expertise to confirm that it is of an acceptable scientific standard, however I have significant reservations, as outlined above.

## References

[1] : Priming by a novel universal influenza vaccine (Multimeric-001)-a gateway for improving immune response in the elderly population. *Vaccine*.2014;32(44) : 10.1016/j.vaccine.2014.08.031 5816-23 10.1016/j.vaccine.2014.08.031 25173483
